# Identifying patients suitable for palliative care - a descriptive analysis of enquiries using a Case Management Process Model approach

**DOI:** 10.1186/1756-0500-5-611

**Published:** 2012-11-01

**Authors:** Ulrike Kuhn, Anne Düsterdiek, Maren Galushko, Christina Dose, Thomas Montag, Christoph Ostgathe, Raymond Voltz

**Affiliations:** 1 Department of Palliative Medicine, University Hospital Cologne, Cologne, Germany; 2 Department of Paediatric Oncology and Haematology, University Hospital Cologne, Cologne, Germany; 3 Division of Palliative Medicine, University Hospital Erlangen, Erlangen, Germany; 4 Centre for Integrated Oncology Cologne/Bonn, Bonn, Germany; 5 Clinical Trials Centre Cologne, Cologne, Germany

**Keywords:** Case management, Palliative care, Identification of patients

## Abstract

**Background:**

In Germany, case management in a palliative care unit was first implemented in 2005 at the Department of Palliative Medicine at the University Hospital Cologne. One of the purposes of this case management is to deal with enquiries from patients and their relatives as well as medical professionals. Using the Case Management Process Model of the Case Management Society of America as a reference, this study analysed (a) how this case management was used by different enquiring groups and (b) how patients were identified for case management and for palliative care services. The first thousand enquiries were analysed considering patient variables, properties of the enquiring persons and the content of the consultations.

**Results:**

Most enquiries to the case management were made by telephone. The majority of requests regarded patients with oncological disease (84.3 %). The largest enquiring group was composed of patients and relatives (40.8 %), followed by internal professionals of the hospital (36.1 %). Most of the enquiring persons asked for a patient’s admission to the palliative care ward (46.4 %). The second most frequent request was for consultation and advice (30.9 %), followed by requests for the palliative home care service (13.3 %). Frequent reasons for actual admissions were the need for the treatment of pain, the presence of symptoms and the need for nursing care. More than half of the enquiries concerning admission to the palliative care ward were followed by an admission.

**Conclusions:**

Case management has been made public among the relevant target groups. Case management as described by the Case Management Process Model helps to identify patients likely to benefit from case management and palliative care services. In addition, with the help of case management palliative patients may be allocated to particular health care services.

## Background

Due to the complex needs of the severely ill, palliative care involves many different health care providers and professionals, e. g. physicians, nurses, social workers, psychologists and chaplains [1-3]. To ensure a high quality and continuity of care according to the patient’s needs, effective coordination of the health care process and the participating services is required [1,4-8]. However, this requirement has not yet been sufficiently met [9]. Case management has been described as a useful organisational approach [10-13] to address the complexity in health care.

According to the Case Management Society of America (CMSA), case management is “a collaborative process which assesses, plans, implements, coordinates, monitors and evaluates options and services to meet an individual’s health needs through communication and available resources to promote quality cost-effective outcomes” ( [14], p.8). According to Powell & Tahan 2010 [15] the CMSA describes six steps of the case management process: client identification and selection, assessment and problem/opportunity identification, development of the case management plan, implementation and coordination of care activities, evaluation of the case management plan and follow-up, and termination of the case management process [15]. In this article, we focus on the first three steps. We analyse the patients’ characteristics and their reasons for the inclusion into the case management process (client identification and selection) as well as the patients’ allocation to different services of palliative care according to their specific needs (assessment and problem/opportunity identification and the first step of the development of the case management plan).

In Germany, the concept of case management has mainly been introduced into the areas of care for the elderly, social and youth welfare, psychiatry, paediatrics and follow-up for breast cancer patients [16-21]. However, it has barely been introduced into palliative care or hospice programs. The first specific case management within palliative care in Germany was implemented at the Department of Palliative Medicine in Cologne on 1 December 2005.

The implementation of case management at the department took place in the course of the universal implementation of case management throughout the whole University Hospital Cologne. Within this hospital, case management is a process of interdisciplinary cooperation in patient care over all departments. The individual medical requirements (aid and support requirements) of a patient are investigated and planned by a case manager in cooperation with the patient and the professionals involved. One important aim is the continuity and quality of care.

### Functions of the case manager at the Department of Palliative Medicine

It is the task of the case manager at the Department of Palliative Medicine to accompany the patient along a care path in his last phase of life according to his needs and across all sectors. The key focus is the coordination of different care options within the hospital (outpatient, wholly or partly inpatient, palliative consultation service). In addition, case management contributes to the networking of general and specialist doctors, nursing services and outpatient hospice services. At the time of the data collection, the Department of Palliative Medicine employed one case manager mainly responsible for the inpatient sector. The service of case management is provided by a nurse with additional training certified by the German Society of Care and Case Management. This person has many years of professional experience as a specialised palliative care nurse at the department and is therefore aware of the structures and all the stakeholders involved in care. In addition, this case manager allocates patients to the palliative home care service but not assumes any other tasks in the outpatient sector. The case manager is the first contact for all internal and external professional groups for information and advice, in-patient admission, requests for the home care service or the palliative consultation service. He identifies patients in need of palliative care and case management. The case manager is also responsible for arranging other care options like a transfer to the hospice or to another ward or department.

Other important fields of work are arranging appointments for patients (e.g. to see a physiotherapist, a psychologist or volunteers) and other administrative tasks like the bed planning in the inpatient setting and the organisation of the patient admission to the ward. There are regular contacts with the patient to ensure the organisation, coordination and documentation of services for the inpatient setting. When the patient is discharged, the case manager organises the further pathway of care (home care, hospice, etc.).

A telephone contact about seven days after discharge serves to check the targets (evaluation).

Besides the case-related support processes, the case manager is also involved in setting up a network. An important task within the system is to participate in the multi-professional team meetings. The case manager is available on workdays from 8:00–16:00. Outside the regular working hours requests by phone are answered by an answering machine.

**Figure 1:**
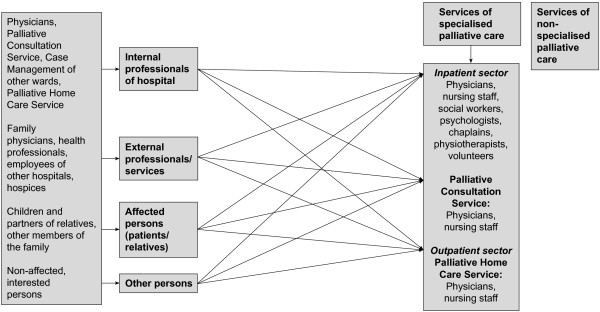
**Palliative care services and the flow of requests before the implementation of case management.** Before implementation of case management, requests were received by various contacts such as physicians or nursing staff. This led to a loss of information as well as a loss of staff members’ time.

Before the implementation of case management, requests were received by various contacts: physicians, nursing staff, etc. (Figure 1). An overview of the problem setting before the implementation of case management at the Department of Palliative Medicine is presented in Table 1 and the specific aims of the implementation are summarised in Table 2.

**Table 1:** Problem setting before the implementation of case management

	
**1. Enquiries and coordination**	All enquiries about admission to the palliative care ward, for the palliative home care service, for advice from the palliative consultation service and for information and advice in general have previously been dealt with by various professional groups such as physicians, nursing staff and the central office. There was no set contact point for external or internal professional groups, or for patients and relatives. This frequently led to misinformation.
**2. Communication**	Communication was not transparent between different care settings as the method of information transmission was not clearly defined.
**3. Care**	Palliative patients receive need-orientated care. Because of frequent changes in symptoms and problem settings, a flexible reaction of all palliative care team members is required. Before the implementation of case management, some services like the transfer management or social services were often not included in due time. As a result, continuity of care could not always be guaranteed.
**4. Networking**	There was insufficient networking between departments in the hospital and between the hospital and other hospitals, general practitioners and specialists, outpatient care services, outpatient hospice services and inpatient hospices.

**Table 2:** Aims of the implementation of case management at the Department of Palliative Medicine

	
**Optimisation of patient care**	The case manager carries out a needs-oriented process accompaniment for the palliative patient (e. g. advice, organisation of aid, contacts with other stakeholders). Palliative care patients from other wards of the University Hospital Cologne and external enquirers gain access to the different services provided by the department. All professional groups receive all relevant information concerning the patients from the case manager.
**Improvement in communication between various professionals**	The aim is to improve communication between the palliative home care service, the palliative ward, the palliative consult service and, if necessary, other external health care services. The means of information transmission shall be made more transparent.
**Releasing some burden from physicians**, **nurses and social workers**	Physicians, nurses and social workers shall be released from administrative and coordinative tasks by the work of the case management.

The aim of this first evaluation following the implementation of case management was to analyse the extent to which the new structure is used by various contact groups within and outside the hospital and what kinds of queries are submitted. To do so, we examined who made the enquiries, the content of the enquiries and how patients were allocated to different palliative care services.

## Methods

### Sample and data collection

This descriptive retrospective study focused on the first thousand enquiries received by case management in the 16 month period after its implementation (23 January 2006 to 25 May 2007). The study was approved by the research ethics committee of the University Hospital Cologne (09–125).

For this explorative approach, a special documentation form was developed which covered the following aspects:

1.Mode of contact (personal or via telephone, e-mail, fax)

2.Diagnosis and basic disease

3.Symptoms

4.Enquiring person

5.Main content of the request

6.Admission to a service of the department

7.Reasons for admission reported by the case management

The routine documentation of the case management was analysed using this documentation form.

## Results

### Mode of contact

Most of the enquiries (85.8%) were dealt with by telephone. The remaining 14.2% consisted of personal enquiries (9.1%), enquiries by fax (4.3%), enquiries by e-mail (0.5%) and other enquiries (0.3%).

### Diagnosis, basic disease and symptoms

It was examined whether the enquiries were based on oncological (84.3%) or non-oncological diseases (4.2%). The most common diseases were tumours of the digestive organs (19.5%), followed by tumours of the respiratory and intrathoracic organs (18.5%). Tumours of the mammary glands (10.1%) were the third most frequent basic diagnosis, followed by tumours of the female genital organs in fourth position (6.2%). Tumours of the urinary organs ranked fifth in this examination (5.6%).

Of the small group of non-oncological diseases, the most common were neurological diseases (2.5%), followed by cardiological diseases (0.5%).

The emphasis on oncological patients is also reflected in the content of requests: oncological diseases were the basis for 96.3% of the enquiries about admission to the palliative care ward, 98.4% of enquiries about the palliative home care, 95.9% of the requests for the palliative consultation service and 90.9% of the enquiries about consultation and advice.

A broad range of symptoms was reported, for example bad health status, physical weakness and shortness of breath.

### Enquiring person

Most enquiries were made by external affected persons (patients and relatives, 40.8%), followed by internal professionals of the hospital (36.1%, Table 3) and external professionals/services (22.5%). The largest subgroup of enquirers among external affected persons was composed of the grown-up children of patients (35%, Table 3). Most enquirers from internal professions were physicians of other departments within the hospital (46%, Table 3).

**Table 3:** Enquiring persons

**Enquiring persons**	**Enquiries**	**Enquiring persons**	**Enquiries**
**External affected persons**	40.8%	Children	35.0%
		Partners	23.5%
		The patient him-/herself	16.9%
		Other family members	15.9%
		Friends and acquaintances	6.9%
		Other persons	1.8%
**Internal professionals of hospital**	36.1%	Physicians	46.0%
		Palliative consultation service	19.1%
		Case Management other wards	12.2%
		Palliative home care service	6.4%
		Nursing staff	5.5%
		Transfer management of medical care	2.8%
		Social workers	1.7%
		Centre for Integrated Oncology (CIO) Köln Bonn	5.5%
		Other internal professionals	0.8%
**External professionals**/**services**	22.5 %	Consultants	56.4%
		Employees of other hospitals	26.2%
		Other services	11.6%
		Out-patient care services of hospices	5.8%
**Other persons**	0.6 %		
	N=1000		

### Main contents of the requests to the case management

The contents of the requests covered admission to the palliative care ward (46.4%), consultation and advice (30.9%), the palliative home care service (13.3%) and the palliative consultation service (8.1%). Differences among the groups were found regarding the distribution of the enquirers (Table 4).

**Table 4:** Distribution of the contents of the requests among the enquiring groups

**Enquiring persons**	**Main content of request**				
	**Admission to palliative care ward**	**Palliative home care service**	**Palliative consultation service**	**Consultation and advice**	**Other contents**
Internal professionals of the hospital	220	48	68	22	3
	(47.4%)	(36.1%)	(84.0%)	(7.1%)	(23.1%)
External professionals/services	130	24	1	68	2
	(28.0%)	(18.0%)	(1.2%)	(22.0%)	(15.4%)
External affected persons	113	60	12	215	8
	(24.4%)	(45.1%)	(14.8%)	(69.6%)	(61.5%)
Other persons	1	1	0	4	0
	(0.2%)	(0.8%)	(0.0%)	(1.3%)	(0.0%)
	n = 464	n = 133	n = 81	n = 309	n = 13
	(100 %)	(100 %)	(100 %)	(100 %)	(100 %)

### Admission to a service of the department

Following an enquiry about admission to the palliative care ward (n = 464) more than half of the patients were actually admitted to the ward (62.3%). 9.8% of the patients who had initially made an enquiry about the palliative home care service (n = 133) were admitted to the ward. 39.8% of the enquiries about palliative home care service (n = 133) were processed through the desired service. 3.6% of enquirers who had initially asked for consultation and advice (n = 309) were admitted to the ward and 0.3% (n = 308) were admitted to the palliative home care service.

### Reasons for admission

The case manager decides about the admission to a palliative service of the department. For this study, the reasons for admission to the palliative care ward, palliative home care service or palliative consultation service were reported by the case management. For each admission, one or two main reasons were documented. The most frequently mentioned reason for admission to the palliative care ward and to the palliative consultation service was the treatment of pain and other symptoms (88.5% and 87.9%, respectively). This was also an important reason for admission to the palliative home care service (35.0%). For 88.3% of admissions to the palliative home care service the need for nursing care was the main objective. Compared to admissions to the palliative home care service, the need for psychosocial support was mentioned more often in connection with admissions concerning the inpatient sector (6.9% and 8.6%, respectively).

## Discussion

This study provides an overview of the first one thousand enquiries made to a newly implemented case management based on the Case Management Process Model.

**Figure 2:**
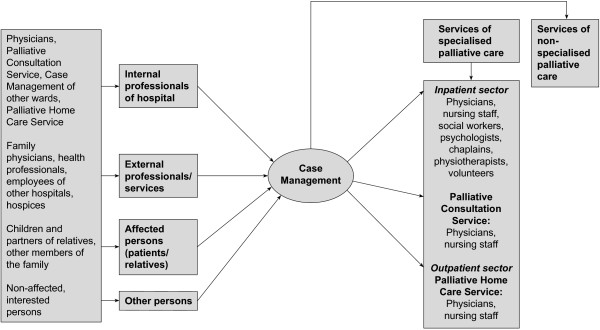
**The flow of requests after the implementation of case management.** By establishing case management, a central contact point for all enquiries regarding palliative care was created. Questions, advice and information are now processed centrally by the case manager. Only in cases of specific issues (for example, medical or legal questions) the requests are forwarded to the responsible team members.

The aim of the study was to examine the extent to which this approach is used by various contact groups within and outside the hospital and what queries are submitted. By breaking down different enquiring groups, it can be shown that the case management as a central contact point (Figure 2) is well known inside and outside the hospital and that different types of people actually use this service. This shows that the implementation of case management has been made public among the relevant target groups. This is an important prerequisite for the successful implementation of case management [22].

In order to achieve this awareness, at the beginning of the process of implementation a lot of publicity was generated. Flyers were distributed both within and outside the University Hospital. These explained the concept of care, the target groups and the functions of case management at the Department of Palliative Medicine, and also contained some contact information. The concept of case management and the implementation process were also introduced and explained at palliative medicine events with presentations, in reports and at symposia at conferences. There was a clear definition of the tasks, responsibilities and authorities of the case manager as a part of the multiprofessional team in advance. The results regarding the mode of contact indirectly indicate effective public relations related to the advertisement of the availability of the case manager by telephone. Stafford and Berra [23] and Vanderplasschen [24] considered the easy access to case managers by phone as an important facilitator of effective case management.

Furthermore, we were interested in the criteria for advisory, allocation and coordination activity of case management and how the criteria are dealt with.

The Case Management Process Model [15] describes the identification and selection of patients who might benefit from case management services as the first step of case management. The specific case management examined here also considers the allocation to other services of palliative care. For the success of this process the case manager has to consider information about symptoms, the burden of symptoms, etc. Depending on these, the case manager has to make a decision about the admission to case management and a palliative care service. In consideration of the reasons for admission named by the case manager, this study indicates that the symptoms are the decisive selection criteria for the allocation to specific areas of medical care. According to Wissert [25] the description and determination of such criteria is important for a successful selection process. The selection process was also covered in a study in an urban hospital in the USA, in which two advanced practice nurses acting as case managers developed an intake needs assessment questionnaire for an initial screening of patients. Just as in our study, the evaluation of physical symptoms and psychosocial issues and the need for advance care planning played an important role. These and others were so-called “palliative care triggers” which served as starting points for referral to palliative facilities and services (‘hospice eligible, requiring relief from symptoms’) [26].

In this study, the fact that more than half of the enquiries concerning the palliative care ward and approximately 40% of those referring to the palliative home care service were followed by an admission to the requested service additionally shows that a selection process had taken place. One can conclude that the case manager decides which service is appropriate.

A wide range of symptoms related to incurable or progressive diseases was documented. It became apparent that in the field of palliative care case managers must be able to recognise complex pain and symptom issues.

The need for psychosocial support as the third most frequent reason for admission to the palliative care ward and palliative consultation service was an interesting result because it shows the need for interdisciplinary palliative care. Case managers have to analyse the psychosocial situation of a patient to enable targeted access to required services [25]. One can suppose that the case manager also has to consider further criteria such as, for example, the patients’ spiritual background and aspects of quality of life. The complex needs of palliative patients require a wide range of skills on the part of the case manager [27,28]. To meet these requirements a special profile of competence is needed [23,29].

This investigation shows the great demand for consultation and advice among external affected persons. This aspect is already considered as a special feature of the case management model at the University Hospital Cologne [30]. The large number of enquiries concerning consultation and advice indicates that there is still room for improvement.

This study is unable to consider enquiries that did not reach the case manager, especially outside the service hours. In addition, there is no evidence for the quality of the handling of the requests. As there is no standardised follow-up considering with the enquiring persons (professionals and patients) the impact and effectiveness of consultation and advice on the patient level cannot be assessed properly. Further research should focus on assessing patient outcomes and measuring quality of life, symptom intensity and patients’ wellbeing [31].

Another open question is the level of satisfaction of the enquiring groups with the case manager’s performance as well as the case management’s acceptability. In order to investigate this, the use of qualitative research methods, for example in-depth interviews and focus groups with the users and providers of the service, is conceivable. These methods could also reveal information about the perceived disadvantages of the service. In addition, a comparison of the data presented here with data measured at a second or third time would be interesting as a means of observing the effects of the process of change on enquiring groups and the contents of enquiries.

In addition, the study does not consider the further course of medical care processes.

The ability to generalise these findings for other settings is limited because the results pertain to the specific situation at the Department of Palliative Medicine at the University Hospital Cologne. A further determination of the criteria that the case manager uses to identify patients in need of case management and palliative services is required. This could be a useful approach to an advancement of the case management process in the field of palliative care.

We feel overall that the case management service itself is accessible and that it facilitates access to other services and providers in the community. We are convinced that this service will result in an improvement of access to palliative care in the examined region. It is likely to provide team coordination to the hospital staff and enable patients to make smooth transitions between care settings. The results of this study are important to decision-makers who are interested in improving palliative care in Germany.

## Conclusions

Case management has been made public among the relevant target groups and is widely known and used locally. A case management structure as described by the Case Management Process Model forms an appropriate approach to identify patients who might benefit from this service in the field of palliative care. In addition, it helps to ensure their allocation to suitable health care services.

### Availability of supporting data

In accordance with the guidelines of the research ethics committee of the University Hospital Cologne, it is not possible to include a data set here.

## Competing interests

The authors declare that they have no competing interests.

## Authors’ contributions

UK drafted the manuscript and was responsible for the analysis and interpretation of data. AD made substantial contributions to the conception and design of the study and was involved in the acquisition, analysis and interpretation of data. MG revised the manuscript critically for important content and participated in the design of the study. CD performed the statistical analysis and helped to draft the manuscript. TM conceived of the study and coordinated and participated in its design. CO revised the manuscript, made contributions to the conception and design of the study and was involved in the acquisition of the data. RV participated in the interpretation of the data and helped to draft the manuscript. All authors read and approved the final manuscript.
